# Phosphatase regulation in cell division: With emphasis on PP2A-B56

**DOI:** 10.1016/j.mocell.2025.100255

**Published:** 2025-07-18

**Authors:** Junsoo Oh, Yeseul Park, Shinae Park, Og-Geum Woo, Jae-Hoon Lee, Jung-Shin Lee, Taekyung Kim

**Affiliations:** 1 Department of Biology Education, Pusan National University, Busan 26241, Republic of Korea; 2 Department of Molecular Bioscience, College of Biomedical Science, Kangwon National University, 1 Kangwondaehak-gil, Chuncheon 24341, Republic of Korea

**Keywords:** Chromosome, LxxIxE motif, Meiosis, Mitosis, Protein phosphatase 2A-B56

## Abstract

Protein phosphatase 2A-B56 (PP2A-B56) is a key regulator of mitosis, playing an essential role in maintaining chromosomal stability and ensuring the fidelity of cell division. As a component of the PP2A holoenzyme, the B56 regulatory subunit confers substrate specificity, primarily through interactions with the conserved LxxIxE motif on target proteins. This review highlights the molecular mechanisms by which PP2A-B56 regulates key processes in cell division, including chromosome cohesion and condensation, kinetochore-microtubule attachment, spindle assembly checkpoint silencing, and activation of the anaphase-promoting complex/cyclosome. In meiosis, PP2A-B56 safeguards centromeric cohesion and facilitates the transition between divisions, with recruitment strategies that differ across species. Recent studies also emphasize its role in protecting oocyte quality and fertility by maintaining chromosomal stability. Furthermore, the competition among multiple LxxIxE-containing substrates for PP2A-B56 binding introduces an additional layer of temporal and spatial regulation. Finally, we discuss how perturbations in PP2A-B56 activity contribute to chromosomal instability and tumorigenesis. Understanding of PP2A-B56's substrate recognition and regulatory dynamics provides a framework for therapeutic targeting in disorders involving defective cell division.

## INTRODUCTION

**Fig. 1:**
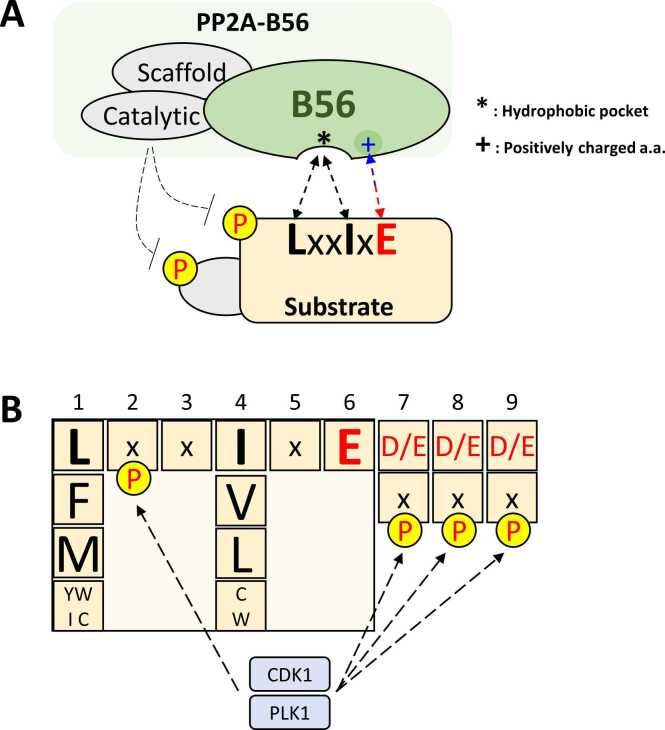
Substrate recognition of PP2A-B56 through binding of B56 subunit to the LxxIxE motif on substrates. (A) The B56 subunit of PP2A-B56 recognizes the LxxIxE motif on substrates to remove phosphorylation on the substrate and its interacting partners. Hydrophobic residues including L and I, and negatively charged residues including E within the LxxIxE motif are bound to the hydrophobic pocket and positively charged residues, respectively, in the B56 subunit of PP2A-B56. (B) The molecular properties inside and around the LxxIxE motif on substrates influence binding to the PP2A-B56. The first or fourth position of the LxxIxE motif often contains some degenerate residues that have a decreased binding affinity to B56. The presence of negatively charged amino acids within 3 amino acids after the LxxIxE motif on the substrate improves binding to the PP2A-B56. Additionally, binding to the PP2A-B56 is enhanced by the addition of a negative charge by CDK1 or PLK1-dependent phosphorylation on the second residue inside the LxxIxE motif or residues within 3 amino acids after the LxxIxE motif.

Reversible protein phosphorylation plays a pivotal role in regulating numerous essential biological processes across all living organisms. This dynamic process involves phosphorylation and dephosphorylation, catalyzed by protein kinases and protein phosphatases, respectively. Protein phosphatase 2A (PP2A) is a major Ser/Thr phosphatase and one of the most abundant phosphatases in cells, forming a heterotrimeric complex comprising a scaffolding A subunit, a regulatory B subunit, and a catalytic C subunit. The catalytic subunit is responsible for enzymatic activity, while the scaffolding subunit anchors both the catalytic and regulatory subunits to form a functional holocomplex. PP2A complexes are categorized based on their regulatory B-subunit families: B55 (B), B56 (B′, PR61), PR72 (B″), and PR93 (B‴). Among these, the PP2A-B56 complex has garnered significant attention due to its interactions with key cell cycle regulators (Barr et al., 2011, Wlodarchak and Xing, 2016, Zhang et al., 2024) (Fig. 1A).

The B56 subunit, a critical member of the B family, confers substrate specificity to the PP2A complex during mitosis. This specificity is dictated by the distinct isoforms of the B56 subunit—B56α, B56*β*, B56γ, B56*δ*, and B56*ε*—each of which preferentially interacts with specific substrates at various stages of the cell cycle, playing vital roles in this process (Barr et al., 2011, Zhang et al., 2024). Despite extensive research on the targeting motifs of protein kinases, the mechanisms by which phosphatases, particularly PP2A-B56, recognize their substrates remain less understood. The identification of a conserved motif that specifically binds to PP2A-B56 has shed light on its roles in diverse cellular processes (Hertz et al., 2016).

This review provides a comprehensive overview of the regulatory roles of PP2A-B56 in both mitosis and meiosis. We highlight how PP2A-B56 modulates critical processes, including chromosome cohesion and condensation, kinetochore-microtubule attachment, spindle assembly checkpoint (SAC) silencing, and activation of the anaphase-promoting complex/cyclosome (APC/C). Furthermore, we emphasize the importance of PP2A-B56 in meiotic chromosome segregation and its emerging implications in human fertility. Particular attention is given to how competition among multiple LxxIxE-containing substrates fine-tunes PP2A-B56's activity and specificity. Finally, we discuss the clinical relevance of PP2A-B56 in disease, including its tumor-suppressive function and potential as a therapeutic target. This review highlights recent structural, biochemical, and cell biological studies to provide an integrated perspective on the regulatory roles of this essential phosphatase.

## SUBSTRATE RECOGNITION OF PP2A-B56

PP2A-B56 identifies its substrates through the regulatory B subunit (Fig. 1A). Analyzing the sequences of KIF4A, GEF-H1, BubR1, and Repo-Man involved in B56 binding has identified a potential B56-binding motif consisting of 6 amino acids (Hertz et al., 2016). Three key positions within this motif—positions 1, 4, and 6—were found to be critical for binding. The conserved short linear motif, referred to as the LxxIxE motif, is characterized by leucine (L) at position 1, isoleucine (I) at position 4, and glutamate (E) at position 6, with “x” denoting any amino acid. This motif facilitates substrate interaction with the B56 subunit through multiple residue-specific interactions (Fig. 1). The importance of this motif was further validated by alanine scanning mutagenesis of LxxIxE motif of the KIF4A peptide. Binding assays using immunopurified YFP-B56⍺ confirmed that the conserved LxxIxE motif is essential for the interaction between KIF4A and B56. Hydrophobic residues, such as leucine and isoleucine, within the LxxIxE motif, are accommodated by a hydrophobic pocket on the B56 subunit, forming stable hydrophobic interactions. Additionally, charge-based interactions play a crucial role, as the negatively charged glutamate at the end of the motif interacts electrostatically with positively charged residues on the surface of the B56 subunit (Fig. 1B).

The binding affinity of PP2A-B56 to its substrates is influenced by the local charge distribution surrounding the LxxIxE motif and the structural properties of the B56-binding pocket. Negatively charged residues located within 3 amino acids of the LxxIxE motif enhance substrate binding by strengthening interactions with the basic residues in the B56 pocket (Fig. 1B). Conversely, the conserved acidic surface near the B56 pocket is critical for binding to basic regions of certain substrates, such as KIF4A and Aim1 (Hertz et al., 2016).

Phosphorylation near or within the LxxIxE motif further enhances substrate binding to PP2A-B56 (Fig. 1B). Substrates with phosphorylated serine or threonine residues either in the second position or within 3 amino acids of the LxxIxE motif peptides of Emi1, RacGAP1, KIF14A, and GEF-H1 exhibit stronger interactions with PP2A-B56. Similar to negatively charged residues, phosphorylation adds negative charges and strengthens the electrostatic interactions with the positively charged regions of PP2A-B56 (Hertz et al., 2016). This phosphorylation-driven enhancement of substrate affinity not only ensures precise targeting of substrate but also increases the efficiency of dephosphorylation of phosphorylated substrates.

Key residues within the LxxIxE motif, such as the initial leucine and fourth isoleucine, often exhibit some degree of variability. Intriguingly, replacing these residues with leucine or isoleucine, respectively, has been shown to strengthen binding interactions. For instance, FOXO3, which contains methionine at the first position of its motif (**M**QT**I**Q**E**), exhibits improved binding to PP2A-B56 when this methionine is substituted with leucine (Hertz et al., 2016). Enhanced binding correlates with increased substrate dephosphorylation, highlighting the functional importance of these substitutions.

In conclusion, the degeneracy of the LxxIxE motif among PP2A-B56 substrates introduces an additional layer of regulation, allowing fine-tuning of the phosphorylation-dephosphorylation balance (Fig. 1A). This variability underscores the adaptability and specificity of PP2A-B56 in cellular signaling processes.

## PP2A-B56 FUNCTIONS IN MITOSIS

Mitosis, a critical process in cell division, ensures the equal distribution of chromosomes into 2 genetically identical daughter cells. PP2A-B56 plays an essential role in regulating various steps of mitosis by modulating the phosphorylation status of key proteins (Barr et al., 2011, Wlodarchak and Xing, 2016, Zhang et al., 2024) (Table 1). Specifically, PP2A-B56 targets the condensin and cohesin complexes, ensuring accurate chromosome condensation and cohesion (Barr et al., 2011, Touati et al., 2019, Ueki et al., 2021, Zhang et al., 2024). Additionally, it dephosphorylates spindle-associated proteins, such as KIF4A, to facilitate proper spindle assembly and functionality (Cutts et al., 2025, Ueki et al., 2021).

**Table 1:** Summary of representative PP2A-B56 interactors

Interactor	LxxIxE motif	Functional context
BubR1	Yes (phospho-dependent)	SAC silencing, kinetochore targeting
KIF4A	Yes (phospho-dependent)	Central spindle regulation
APC1	Yes (phospho-dependent)	Anaphase onset via APC/C
Sgo1	No	Cohesion protection in mitosis
Sgo2	No	Cohesion protection in mitosis, meiosis I, and meiosis II
BUB-1	Yes (only in *Caenorhabditis elegans*)	Cohesion protection in meiosis I

The table includes selected interactors with known or predicted LxxIxE motifs, and annotated functional roles in mitosis, meiosis. This is a representative, nonexhaustive list intended to aid comparison and highlight common regulatory themes.

APC/C, anaphase-promoting complex/cyclosome; SAC, spindle assembly checkpoint.

During mitosis, the SAC monitors the attachment of chromosomes to the mitotic spindle, preventing premature anaphase onset. PP2A-B56 contributes to SAC regulation by dephosphorylating and stabilizing key checkpoint proteins, including BubR1, Cdc20, and Mad2 (Lara-Gonzalez et al., 2021). These proteins are essential for maintaining checkpoint integrity, thereby safeguarding accurate chromosome segregation.

Furthermore, PP2A-B56 plays a pivotal role in the mitotic exit by regulating the dephosphorylation of critical proteins such as Cdc14 and APC1 (Fujimitsu and Yamano, 2020, Touati et al., 2019). This regulation facilitates the transition from mitosis to cytokinesis, ensuring the precise division of the cytoplasm and the formation of 2 daughter cells.

### CHROMOSOME COHESION AND CONDENSATION

PP2A-B56 plays a pivotal role in maintaining proper chromosome condensation and cohesion by modulating the phosphorylation states of condensin and cohesin complexes. Localization of PP2A-B56 to centromeres is mediated through its interaction with shugoshin proteins (Sgo1 and Sgo2) (Kitajima et al., 2006, Ueki et al., 2021). Although Sgo1 and Sgo2 do not possess the LxxIxE motif, human Sgo1 (hSgo1) facilitates the recruitment of PP2A-B56α/*ε* to centromeres (Ueki et al., 2021). This interaction safeguards cohesin complexes during prophase by antagonizing mitotic kinase activity and mitigating wings apart-like protein homolog-mediated cohesin removal. Interestingly, it has been demonstrated that while hSgo1 lacks the LxxIxE motif, LxxIxE peptides can compete with hSgo1 for binding to PP2A-B56⍺ purified from prometaphase-arrested cells. Mutations in the LxxIxE-binding pocket of PP2A-B56γ disrupt its interaction with hSgo1 and compromise cohesin protection, suggesting that the LxxIxE-binding pocket is critical for this function. While the C-terminal HEAT repeats of Sgo1 were previously proposed to interact with B56, mutations in this region did not affect cohesin protection, underscoring the importance of the interaction between hSgo1 and the LxxIxE-binding pocket of PP2A-B56 (Ueki et al., 2021).

KIF4A is a kinesin that binds to chromosomes and plays a crucial role in maintaining normal chromosome architecture and regulating the anaphase spindle during cell division (Cutts et al., 2025, Mazumdar et al., 2004, Wang et al., 2020). Depletion of KIF4A and its redundant protein hKid causes significant mitotic delay, with multiple unaligned chromosomes (Wang et al., 2020). KIF4A contains the LxxIxE motif, which interacts with B56, and it also possesses a basic patch essential for binding to chromosomes via condensin I (Hertz et al., 2016, Wang et al., 2020). The binding of B56 or condensin I to KIF4A is mutually exclusive, and mutation of the basic patch disrupts KIF4A's ability to bind chromosomes. This also impaired the dephosphorylation of KIF4A by B56, a critical process for regulating the anaphase spindle (Wang et al., 2020). This suggests a functional interplay between its role in mitotic regulation and its recognition by PP2A-B56.

### KINETOCHORE-MICROTUBULE ATTACHMENTS AND THE SAC SILENCING

**Fig. 2:**
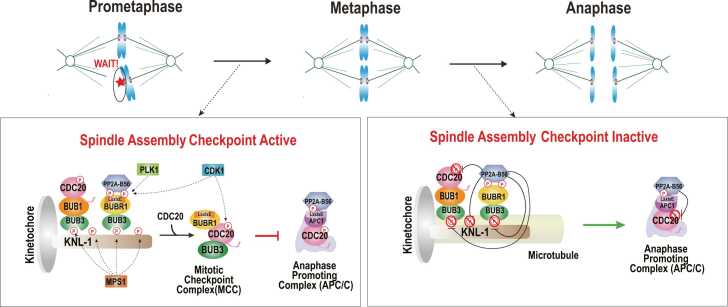
The function of PP2A-B56 in mitosis. A schematic representation of PP2A-B56 localization and function during mitosis. PP2A-B56 is recruited to kinetochores through its interaction with BubR1, which contains an LxxIxE motif. The phosphorylation of this motif by PLK1 or Cdk1 enhances PP2A-B56 binding. When kinetochores are not properly attached to spindle microtubules, the spindle assembly checkpoint (SAC) is activated, delaying anaphase onset. During this phase, phosphorylation of Cdc20 prevents its binding to the anaphase-promoting complex/cyclosome (APC/C), maintaining checkpoint activation. In addition, the localization of the PP2A-B56 by BubR1 contributes to the proper chromosome alignment. Once all kinetochores are properly attached to spindle microtubules, the SAC is silenced, allowing cells to progress into anaphase. PP2A-B56 contributes to SAC silencing by dephosphorylating KNL1, leading to the delocalization of the Bub1/Bub3 complex. Additionally, PP2A-B56 is recruited to the Apc1 subunit of APC/C through its LxxIxE motif, where it dephosphorylates Cdc20, promoting its binding to APC/C and triggering anaphase onset.

BubR1 is a key protein responsible for recruiting PP2A-B56 to kinetochores, where it plays a crucial role in regulating chromosome congression. This is primarily achieved through the interaction between PP2A-B56 and BubR1, which antagonizes Aurora B kinase activity and ensures proper chromosome alignment during metaphase. The kinetochore attachment regulatory domain of BubR1 interacts with PP2A-B56 via the LxxIxE motif (Fig. 2). Moreover, phosphorylation of nearby sites, including Ser670, Ser676, and Thr680, by Cdk1 and Plk1 at unattached or tension-less kinetochores enhances binding to B56 (Espert et al., 2014, Foley et al., 2011, Kruse et al., 2013, Nijenhuis et al., 2014, Suijkerbuijk et al., 2012, Xu et al., 2013). This interaction is essential for proper chromosome segregation. Additionally, it was suggested that the LxxIxE motif of BubR1 is sufficient for the interaction with B56 (Wang et al., 2016).

Beyond its role in kinetochore attachment, BubR1 is a critical component of the SAC, a safeguard mechanism activated at unattached kinetochores. The SAC depends on the phosphorylation of Knl1 MELT repeats by Mps1 kinase, which recruits SAC proteins, including BubR1. BubR1, along with Bub3 and Cdc20, forms the mitotic checkpoint complex, which inhibits the APC/C, thereby delaying anaphase onset (Fig. 2) (Lara-Gonzalez et al., 2012, London et al., 2012, Shepperd et al., 2012, Yamagishi et al., 2012).

The association between PP2A-B56γ and BubR1 has also been implicated in SAC efficiency by stabilizing BubR1. Depletion of B56γ leads to BubR1 degradation throughout mitosis, particularly in nocodazole-treated cells, resulting in abnormal chromosome segregation—a hallmark of reduced SAC efficiency (Varadkar et al., 2017). While various B56 isoforms (B56⍺, B56γ, and B56*ε*) localize to chromosomes, polar microtubules, and mitotic poles, B56γ appears to have a specific role in BubR1 stabilization (Vallardi et al., 2019).

BubR1 also contributes to SAC silencing by recruiting PP2A-B56, which dephosphorylates the Knl1 MELT repeats (Fig. 2) (Cordeiro et al., 2020, Foley et al., 2011, Kruse et al., 2013, Smith et al., 2019, Suijkerbuijk et al., 2012, Xu et al., 2013). The removal of BubR1 from kinetochores disrupts Knl1 MELT dephosphorylation, delaying mitotic exit following Mps1 inhibition in nocodazole-treated cells (Cordeiro et al., 2020, Gama Braga et al., 2020). Furthermore, mutations in the LxxIxE motif of BubR1 attenuate SAC silencing, underscoring its critical role in this process (Xu et al., 2013).

### APC/C ACTIVATION AND ANAPHASE ENTRY

PP2A-B56 also promotes entering anaphase by promoting the association of Cdc20 with APC/C. Cdc20 contains the Cdk1-dependent phosphorylation of 3 N-terminal threonine residues near the C-box and dephosphorylation of these sites was shown to promote association of Cdc20 to APC/C to enter anaphase (Houston et al., 2023, Kim et al., 2017, Labit et al., 2012). Apc1 is one of the components of the APC/C that contains the LxxIxE in the disordered loop domain. In the *Xenopus laevis* extracts, the APC1-loop domain showed the binding to the B56γ, but this interaction was not observed in the interphase that has low Cdk activity (Fujimitsu and Yamano, 2020). In addition, nonphosphorylatable mutant of Apc1-loop^500^ abolished the binding to the B56γ, suggesting that the binding of B56 to Apc1-loop^500^ depends on phosphorylation of the loop domain by Cdk (Fig. 2).

The APC/C that contains the mutation of the LxxIxE motif in Apc1-loop^500^ showed lower affinity for Cdc20 than the WT APC/C, indicating that Apc1-loop^500^ plays a role in formation of APC/C-Cdc20 complex in anaphase, and B56γ may have a role in dephosphorylating Cdc20 (Fujimitsu and Yamano, 2020). When Cdk1-dependent phosphorylation site of Cdc20 is mutated to the nonphosphorylatable mutant, Cdc20 was able to bind to the APC/C efficiently even though LxxIxE motif in Apc1-loop^500^ is abolished. Altogether, these results suggest that B56 that binds to the APC1 promotes binding of Cdc20 to the APC/C by dephosphorylating the Cdk1 site of the Cdc20 (Fig. 2).

In human cells, depletion of all B56 isoforms together, which includes B56α, B56*β*, B56γ, B56*δ*, and B56*ε,* showed delayed anaphase onset, but excluding B56*β* delayed similar extent as depletion of all B56 isoforms, suggesting that B56*β* is not essential for inducing a delay in anaphase onset (Vallardi et al., 2019). B56 depletion did not alter the stability of the mitotic checkpoint complex, but it reduced the association of APC/C-Cdc20 complex, suggesting that B56 promotes the association of Cdc20 with APC/C to enter anaphase (Lee et al., 2017). In addition, B56 coprecipitated with APC/C subunits, including APC1, APC4, and APC7, independently of BubR1. The mutant of B56α that poorly binds to the LxxIxE motif reduced the binding with Cdc20, APC4, and APC7, suggesting that LxxIxE motif may exist in the APC/C subunits.

In conclusion, B56 that binds to the APC/C independently of the BubR1 promotes entering the anaphase by promoting the association of APC/C and Cdc20.

## ROLE OF PP2A-B56 IN MEIOSIS

During meiosis, 2 successive divisions (meiosis I and II) are processed without an intermediate S phase. PP2A-B56 has critical functions in meiosis, particularly in safeguarding cohesion during the first division and ensuring proper kinetochore function across both divisions (Table 1). However, PP2A-B56 exhibits distinct regulatory behaviors during meiosis. In oocytes, the phosphatase faces the challenge of maintaining cohesion over decades of cell cycle arrest and then coordinating its removal in a stepwise fashion, a process intimately tied to PP2A-B56 action (Keating et al., 2020). Below, we outline the major meiotic roles of PP2A-B56, emphasizing recent findings in various model organisms.

### PROTECTION OF CENTROMERIC COHESION IN MEIOSIS

**Fig. 3:**
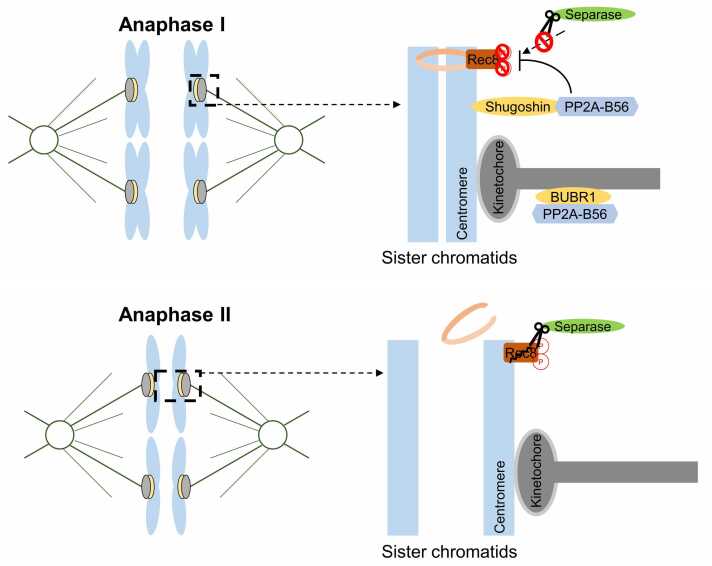
The function of PP2A-B56 in meiosis. A schematic representation of PP2A-B56 localization and function during meiosis. During meiosis I, PP2A-B56 is recruited to centromere and kinetochores through its interaction with Shugoshin and BubR1, respectively, which contain an LxxIxE motif. PP2A-B56 in the centromere dephosphorylates the cohesion component Rec8, thereby preventing cohesion removal by separase. So, the sister chromatids remain tightly bound during anaphase I while homologous chromosomes are segregated. In metaphase II, both Shugoshin and BubR1 are removed, so PP2A-B56 could not bind to centromeres and kinetochores anymore. Phosphorylated Rec8 is cleaved by separase, and the sister chromatids are segregated in anaphase II.

During meiosis I, sister chromatids stay joined at their centromeres while homologous chromosomes segregate. This is achieved by protecting centromeric cohesin from separase during anaphase I (Fig. 3). The protector is the Shugoshin-PP2A-B56 complex. In human oocytes, Shugoshin-2 (Sgo2) is enriched at centromeres and recruits PP2A-B56 to the centromere (Mihalas et al., 2024, Ueki et al., 2021). PP2A-B56 in turn dephosphorylates the cohesin subunit Rec8, keeping Rec8 unphosphorylated at centromeres (Riedel et al., 2006) (Fig. 3). Since separase can only cleave phosphorylated Rec8, this action effectively protects the centromeric cohesin in meiosis I. In mouse oocytes, when PP2A-B56 was absent, sister chromatid prematurely separated, leading to a fatal error that compromises gamete fertility (Tang et al., 2016). *Drosophila* has a single meiosis-specific Shugoshin (MEI-S332) that recruits PP2A (B56 subunits called Wdb/Wrd in flies) to protect centromere cohesion in meiosis I. The knockdown of B56 subunits in oocytes in *Drosophila* causes frequent chromosome mis-segregation due to cohesion loss (Jang et al., 2021). Taken together, PP2A-B56 recruitment by Sgo2 or MEI-S332 is critical for safeguarding centromeric cohesion during meiosis I across species, and its loss results in premature cohesion release and meiotic failure.

After meiosis I, oocytes proceed into meiosis II. Now the challenge is to remove the centromeric cohesin that was preserved through meiosis I, so that sister chromatids can separate in anaphase II. Sgo2 and PP2A-B56 do remain at centromeres in metaphase II oocytes, colocalizing with cohesin that is not removed from meiosis I (Jang et al., 2021). However, once anaphase II is triggered, Sgo2 is removed and PP2A-B56 is no longer centromere-localized, allowing separase to cleave Rec8 at centromeres (Mihalas et al., 2024) (Fig. 3). When PP2A-B56's centromeric signal disappears, Rec8 gets phosphorylated and cleaved, allowing sisters to separate. In conclusion, timely removal of the Sgo2-PP2A-B56 complex from centromeres is a critical switch that enables separase-mediated cleavage of Rec8 and sister chromatid separation during anaphase II, ensuring proper completion of meiosis.

### ROLES IN KINETOCHORE FUNCTION AND CHROMOSOME ALIGNMENT IN MEIOSIS

PP2A-B56 also contributes to proper kinetochore-microtubule interactions in meiosis. During meiosis I, kinetochores of sister chromatids co-orient, which is distinct from mitotic biorientation. In mouse oocytes, PP2A-B56 localizes both to the inner centromere via Sgo2 and outer kinetochore via BubR1 in prometaphase I (Mihalas et al., 2024, Touati et al., 2015) (Fig. 3). The centromeric pool and kinetochore pool may create a spatial gradient of dephosphorylation that accommodates the unique geometry of meiosis I kinetochores. For example, as in mitosis, BubR1-bound PP2A-B56 can stabilize kinetochore-MT attachments by opposing Aurora B/C kinase. Aurora B/C is active in meiotic oocytes to release improper attachments (Yoshida et al., 2015). PP2A-B56 counteracts it once correct attachments form. Consistently, in knockout of PP2A-C in mouse oocytes that lack all PP2A phosphatase activity, chromosomes fail to congress in meiosis I and show unstable attachments (Tang et al., 2016, Yoshida et al., 2015). In *Drosophila,* knockout of PP2A-B56 leads to loss of kinetochore-microtubule attachments and defects in chromosome biorientation (Jang et al., 2021). Thus, across species, PP2A-B56 promotes accurate chromosome alignment and SAC satisfaction in meiosis I by stabilizing attachments once they are appropriate for the meiotic configuration.

### ALTERNATE RECRUITMENT MECHANISMS IN MEIOSIS

A fascinating discovery in meiosis comes from *C. elegans*, which lacks a canonical Shugoshin and whose BubR1 ortholog cannot bind B56. Worm oocytes still require PP2A-B56 for meiotic chromosome segregation, but they recruit it in a novel way. Bel Borja et al. (2020) found that in *C. elegans*, the kinase BUB-1 is the primary factor targeting B56 to chromosomes during meiosis I. BUB-1 contains a newly identified LxxIxE motif that, when phosphorylated, binds B56 and localizes PP2A-B56 to chromosomes in prometaphase I. This PP2A-B56 pool on chromosomes is crucial for proper chromosome congression. When LxxIxE motif in BUB-1 is mutated, PP2A-B56 fails to target, and chromosomes do not align correctly on the meiotic spindle. This finding highlights the versatility of the LxxIxE recruitment mechanism: even in the absence of BubR1 or Sgo, the cell can evolve an alternative recruiter such as Bub1 in this case to bring PP2A-B56 to where it is needed. It also underscores the conserved importance of PP2A-B56 in meiosis, whether via Bub1, BubR1, or Sgo, the oocyte makes sure to harness B56 for both cohesion protection and attachment regulation.

### IMPLICATIONS FOR FERTILITY AND ANEUPLOIDY

The role of PP2A-B56 in female meiosis has direct implications for human fertility. Notably, human oocytes exhibit a remarkably high incidence of aneuploidy compared with sperm or mitotic cells (Jones, 2008, Pacchierotti et al., 2007). Erroneous chromosomal segregation during oocyte meiosis results in gametes with missing or extra chromosomes, a major cause of miscarriages and congenital disorders like Down syndrome. Aneuploidy in oocytes increases dramatically with maternal age, and one of the major causes is the deterioration of cohesin and its protection mechanisms over time. Recent studies in human oocytes have directly linked age-related cohesion loss to Sgo2/PP2A-B56 dysfunction (Mihalas et al., 2024). Oocytes from younger and older women showed that Sgo2 levels at centromeres are significantly reduced in eggs from older women. In many older oocytes, Sgo2 was prematurely lost from the pericentromeric “bridge” that links sister centromeres, leading to weakened cohesion between sister chromatids. This loss of Sgo2 was correlated with increased incidence of separated sister chromatids observed at meiosis II in those oocytes. The study also showed that experimentally depleting Sgo2 during meiosis I by using Mps1 inhibitor caused more single chromatids to appear in meiosis II, directly demonstrating that Sgo2 is needed to preserve cohesion until meiosis II. In conclusion, as women age, the protection of the cohesion by Sgo2/PP2A-B56 fails, which results in increased chromosome mis-segregation. Beyond cohesion, PP2A-B56 may also affect oocyte quality through other pathways, such as maintaining proper spindle assembly via BubR1, or DNA damage responses during the long prophase arrest (Li et al., 2007, Suijkerbuijk et al., 2012). From a clinical perspective, the Sgo2-PP2A-B56 axis can be an intriguing candidate for interventions to improve oocyte quality or prevent age-related infertility. However, any such intervention would require delicately boosting cohesion protection without impeding the eventual release in meiosis II.

## COMPETITIVE BINDING AND REGULATORY TUNING AMONG LXXIXE SUBSTRATES

Given that many different proteins carry the LxxIxE motif, a crucial question is how PP2A-B56 selects or balances among multiple potential binders. Inside a cell, PP2A-B56 holoenzymes are limited in number, so competition for the B56 docking groove can occur between substrates that are present simultaneously. Cells have evolved strategies to coordinate this competition, ensuring that PP2A-B56 engages the correct substrate at the correct moment.

One basic mechanism for prioritization is differential affinity. As discussed, not all LxxIxE motifs bind B56 with equal strength. For example, during early mitosis, BubR1's phosphorylated kinetochore attachment regulatory domain motif might bind B56 with higher affinity than other motifs, thereby sequestering PP2A-B56 at kinetochores until anaphase (Suijkerbuijk et al., 2012). In anaphase, KIF4A’s motif may become relevant in the central spindle region where BubR1 is no longer present, thus redirecting PP2A-B56 to KIF4A (Mazumdar et al., 2004). The cell can modulate these affinities dynamically. The phosphorylation state functions as a regulatory modulator, whereby a low-affinity motif can be converted into a high-affinity one by the addition of phosphate groups such as MPS1 and Cdk1 effectively creating a phospho-dependent “on-switch” for B56 binding (Hertz et al., 2016). Conversely, some motifs might be kept unphosphorylated until their turn is needed. This ensures a temporal sequence in B56-substrate engagement. For instance, Knl1's MELT motifs bind Bub3/Bub1 when phosphorylated by MPS1, but once attached, PP2A-B56 that is recruited by BubR1 dephosphorylates MELT and adjacent sites, which promotes PP1 binding and prevents PP2A-B56 from staying bound (Fig. 2) (Espert et al., 2014). Once PP2A-B56 silences the checkpoint and then is itself removed from attached kinetochores. Such feedback loops illustrate how the cell dynamically regulates the availability of B56 to different partners.

Another mechanism is structural separation of binding interfaces. Certain recruiters such as Sgo1/2 and BubR1 bind B56 at distinct surfaces or contexts, allowing them to bind simultaneously without direct competition. The crystal structure of a BubR1-B56 complex showed that BubR1's LxxIxE binds one face of B56, while Shugoshin can bind another face without steric clash (Wang et al., 2016). This suggests a single PP2A-B56 could interact with both a Shugoshin- and a BubR1-bound substrate at once. Whether such ternary complexes occur in vivo is still being investigated, but at least it means Sgo and BubR1 are not direct competitors for the exact same binding pocket on B56.

In addition to the structural nonoverlap, cells also utilize spatial compartmentalization to limit direct competition between the substrates that share the same binding groove. For example, GEF-H1 and BubR1 would rarely be in the same complex because GEF-H1 localizes to the mitotic spindle until anaphase, whereas BubR1-B56 acts at kinetochores in prometaphase/metaphase (Birkenfeld et al., 2007, Suijkerbuijk et al., 2012). Such spatial separation helps maintain target specificity and prevents mutually exclusive binding events that could otherwise titrate B56 away from essential substrates.

To further understand this competition, modern proteomic and single-cell imaging approaches are starting to reveal when and where each B56-interacting protein actually engages the phosphatase. A remaining challenge is to map the complete “interactome” of PP2A-B56 during the cell cycle and to understand the kinetic preferences. If multiple motifs are present, can PP2A-B56 dephosphorylate several substrates in one vicinity or does it commit to one at a time? Emerging tools like localized optogenetic recruitment of motifs or competitive peptides will help dissect these questions (Allan et al., 2025).

Appreciating the competitive aspect has practical implications. For instance, in cancer therapy, small molecules that disrupt a specific B56-substrate interaction could be a strategy to selectively modulate PP2A activity. Conversely, mimetic peptides that compete for B56 might be used to globally activate kinase signaling for transient periods, which could, for example, push cells past a checkpoint in certain controlled scenarios.

In summary, PP2A-B56 operates at the hub of a network of competing interactions. The cell’s regulatory circuits ensure an orderly queue for B56's attention, primarily through phosphorylation-based switches and scaffolding proteins that create dedicated B56 pools. This competitive tuning is a sophisticated means to integrate multiple signaling pathways, including Cdk1, Plk1, Mps1, and Aurora with the final phosphatase output.

## CONCLUSION AND FUTURE DIRECTIONS

The PP2A-B56 holoenzyme has emerged as a master regulator of chromosome dynamics, unifying the control of mitosis and meiosis through a common mechanism of targeted dephosphorylation. Central to its function is the LxxIxE docking motif, which acts like a postal code directing PP2A-B56 to specific substrates and subcellular locations. By reading this short motif and its phosphorylation modifications, PP2A-B56 can discern when and where to act during cell division. We have seen that B56 subunit isoforms use this mechanism in slightly different ways, partnering with distinct recruiters, such as BubR1, Sgo1/2, and KIF4A to fulfill specialized roles in cohesion protection, kinetochore function, or cytokinesis.

One striking theme is how phosphatase specificity can rival that of kinases. Classically, phosphatases were regarded as blunt enzymes. However, PP2A-B56 clearly shows that specificity is achieved through modular subunits and docking motifs, analogous to how kinases have docking domains or localization signals. This realization opens new avenues for research. For example, can we find small molecules or peptides that mimic the LxxIxE motif to selectively hijack PP2A-B56? Some viral proteins already carry such motifs, suggesting nature’s prototypes for PP2A hijacking (Hertz et al., 2016). Understanding these interactions might inspire therapeutic strategies to manipulate PP2A-B56 activity in diseases.

In cancer biology, PP2A-B56 is generally tumor-suppressive, as it constrains oncogenic phosphorylation such as β-catenin in Wnt signaling or Myc stability (Peris et al., 2023). Indeed, several cancers harbor suppressed PP2A activity via upregulating inhibitors like CIP2A or mutating PP2A subunits (Nader et al., 2019, Pavic et al., 2023). Restoring PP2A-B56 function is thus a promising anticancer strategy. Current efforts include PP2A-activating drugs such as SMAPs that interact with PP2A-A subunit and activate the phosphatase (Nader et al., 2019). A recent structural study provided a blueprint of how CIP2A binds and inhibits B56α, which could guide the design of molecules to block CIP2A-B56 binding and free PP2A to do its tumor-suppressive work (Pavic et al., 2023).

In the context of reproductive medicine, recruitment of PP2A-B56 to the centromere via Sgo2 is a key factor in age-related aneuploidy. Could enhancing Sgo2 or PP2A-B56 activity in oocytes of older individuals reduce aneuploidy rates? This is speculative, as globally increasing PP2A might have other consequences. But perhaps a targeted approach, such as delivering Sgo2 to oocyte centromeres or inhibiting cohesin phosphorylation, could mimic the effect. Any future intervention would need to be tightly controlled to avoid trapping cohesin when it should be removed. Nonetheless, just diagnosing low Sgo2/PP2A-B56 levels could be useful as a marker of oocyte quality.

On the cell biology front, many open questions remain. For example, how is PP2A-B56 itself regulated through the cell cycle? We know kinases like Cdk1/Plk1 target the substrates to modulate binding, but do they also phosphorylate B56 subunits to alter their affinity or localization? Uncovering any “phospho-code” on B56 could add a new layer to the regulation we discussed. An additional consideration is what constraints limit PP2A-B56's activity in vivo. We often think of phosphatases as continuously active, yet clearly their action is restrained until the right moment. Although phosphatases are generally considered constitutively active, their function is clearly temporally regulated. It remains to be determined whether this regulation involves conformational alterations in the holoenzyme or transient inhibition by endogenous competing peptides.

In conclusion, the past decade has vastly expanded our understanding of how PP2A-B56 is targeted and regulated. We now understand that a short linear motif, LxxIxE, is central to how PP2A-B56 controls chromosome segregation. The challenge moving forward is to integrate this molecular detail into a system-level understanding of cell division and to leverage it for human health. By dissecting the “when, where, and how” of PP2A-B56 activity, researchers are uncovering new ways to correct or exploit cell division processes. Future work will aim to map the full landscape of PP2A-B56 interactions and to determine how each interaction influences cellular physiology or pathology. Such knowledge could pave the way for discriminatory targeting of PP2A-B56 holoenzymes. The regulatory roles of PP2A-B56 in mitosis and meiosis illustrate how modular molecular design enables precise control over complex cellular processes. Continued exploration of this system will provide both fundamental insights and practical benefits.

## Author Contributions

**Og-Geum Woo:** Writing – review & editing. **Shinae Park:** Writing – review & editing. **Jung-Shin Lee:** Writing – review & editing, Project administration, Conceptualization. **Jae-Hoon Lee:** Writing – review & editing. **Taekyung Kim:** Writing – review & editing, Writing – original draft, Funding acquisition, Conceptualization. **Junsoo Oh:** Writing – original draft. **Yeseul Park:** Writing – review & editing.

## Declaration of Competing Interest

The authors declare that they have no known competing financial interests or personal relationships that could have appeared to influence the work reported in this paper.
